# Supplementary material for: A Zur-mediated transcriptional regulation of the zinc export system in Pseudomonas aeruginosa
Source: BMC Microbiol. 2023 Jan 9;23:6. doi: 10.1186/s12866-022-02750-4 (PMC9827704; doi:10.1186/s12866-022-02750-4)
Supplement: Supplementary file 1 — Additional file 1: Figure S1. A) DNAse I footprinting of czcR promoter (template strand) in the absence or presence or of 1 µM Zur protein. DNA fragments were analyzed by capillary electrophoresis. The two Zur boxes are indicated by the numbers 1 and 2 in red. The sequencing reaction is visible below the figure (seq1) and the sequence with the two boxes indicated in red is shown below the figure. B) Transcription start site (+1) of czcR and czcC determined by 5’RACE. The CzcR box, according to (1) is indicated in the box and the -35, -10 sequences were determined in silico using BPROM program (2). [file 12866_2022_2750_MOESM1_ESM.pdf]

Figure S1

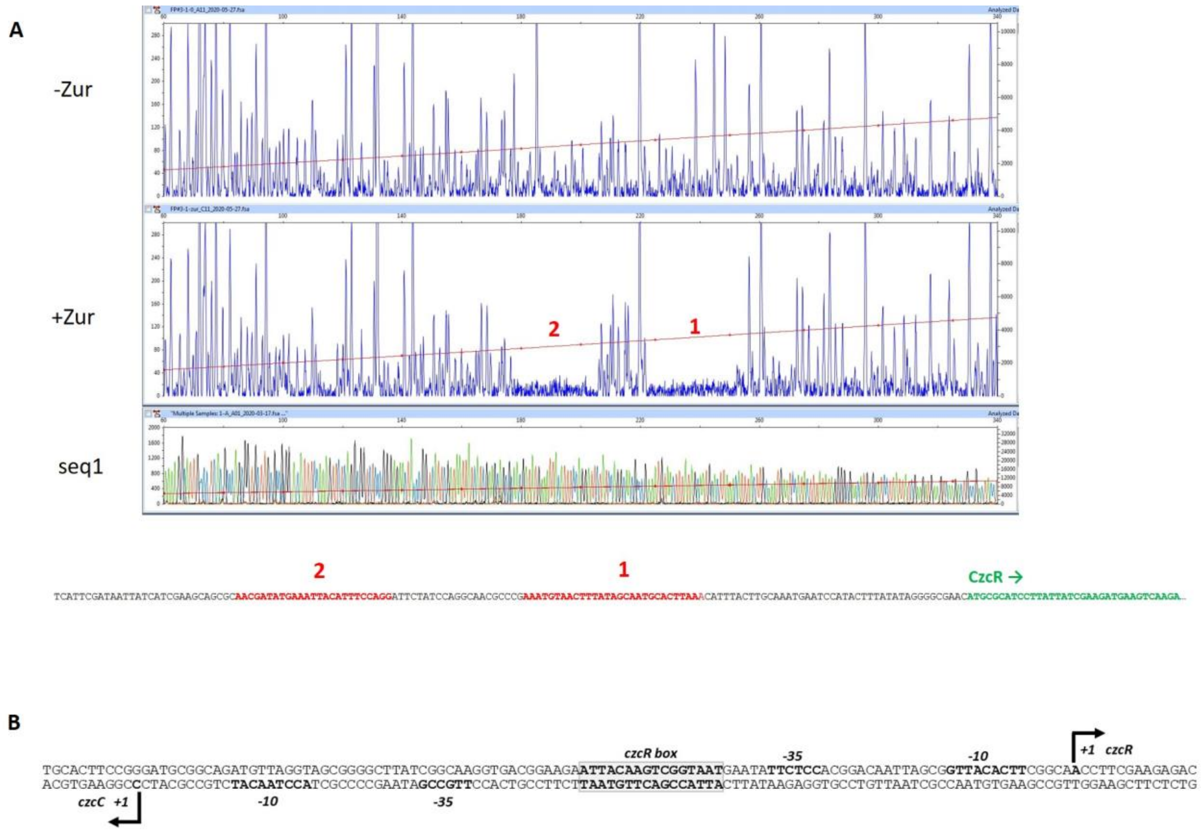

Figure S1. A) DNAse I footprinting of *czcR* promoter (template strand) in the absence or presence or of 1 µM Zur protein. DNA fragments were analyzed by capillary electrophoresis. The two Zur boxes are indicated by the numbers 1 and 2 in red. The sequencing reaction is visible below the figure (seq1) and the sequence with the two boxes indicated in red is shown below the figure. **B)** Transcription start site (+1) of *czcR* and *czcC* determined by 5'RACE. The CzcR box, according to (1) is indicated in the box and the -35, -10 sequences were determined *in silico* using BPROM program (2).

1. Fan, K., Cao, Q. and Lan, L. (2021) Genome-Wide Mapping Reveals Complex Regulatory Activities of BfmR in Pseudomonas aeruginosa. *Microorganisms*, **9**.
2. Solovyev, V. and Salamov, A. (2011) In Li, R. W. (ed.), *Metagenomics and its Applications in Agriculture, Biomedicine and Environmental Studies*. Nova Science Publishers, pp. 61-78.
