# Supplementary material for: A Zur-mediated transcriptional regulation of the zinc export system in Pseudomonas aeruginosa
Source: BMC Microbiol. 2023 Jan 9;23:6. doi: 10.1186/s12866-022-02750-4 (PMC9827704; doi:10.1186/s12866-022-02750-4)
Supplement: Supplementary file 2 — Additional file 2: Figure S2. Growth curves of the wt PAO1 strain, the Δzur mutant and the ΔzurΔznuB double mutant without or in presence of 2 mM ZnCl2 (Zn). Standard deviations of the triplicates are indicated. [file 12866_2022_2750_MOESM2_ESM.pdf]

**Figure S2**

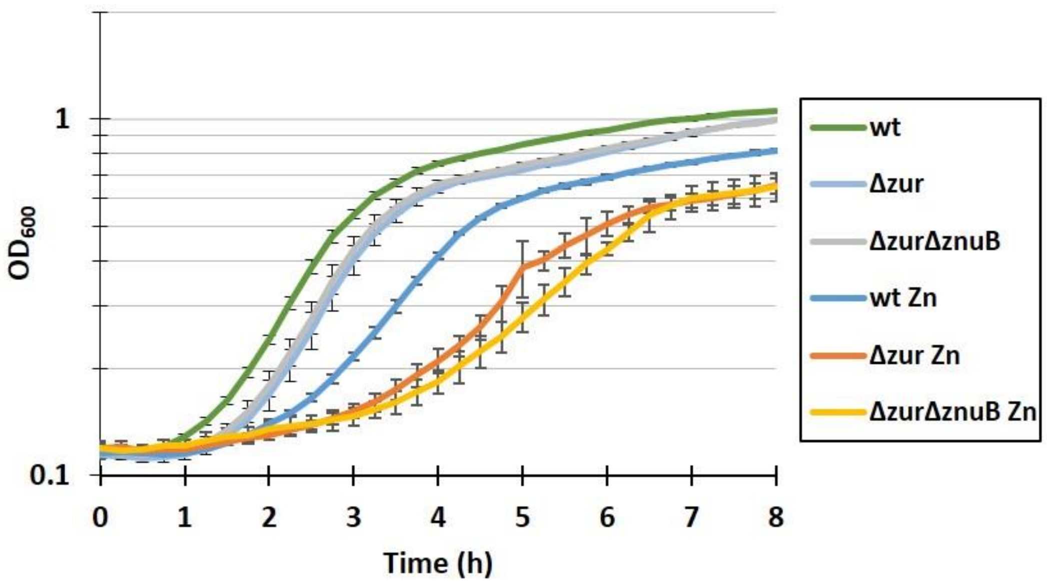


**Figure S2.** Growth curves of the wt PAO1 strain, the Δ*zur* mutant and the Δ*zur*Δ*znuB* double mutant without or in presence of 2 mM $ZnCl_2$ (Zn). Standard deviations of the triplicates are indicated.
