# Supplementary material for: A Zur-mediated transcriptional regulation of the zinc export system in Pseudomonas aeruginosa
Source: BMC Microbiol. 2023 Jan 9;23:6. doi: 10.1186/s12866-022-02750-4 (PMC9827704; doi:10.1186/s12866-022-02750-4)
Supplement: Supplementary file 3 — Additional file 3: Figure S3. GFP promoter fusions containing 542 bp upstream of the czcC DNA regionincluding either the wt or the mutated Zur boxes (mut1+2). Fusions were transformed into the wt PAO1 strain. Cultures were incubated at 37°C with shaking in a microplate reader and fluorescence measurement was performed every 15 min after the addition of 2mM ZnCl2, or without the addition of Zn, as indicated. The fluorescence values are normalized with cell density. Standard deviations of the triplicates are indicated. [file 12866_2022_2750_MOESM3_ESM.pdf]

Figure S3

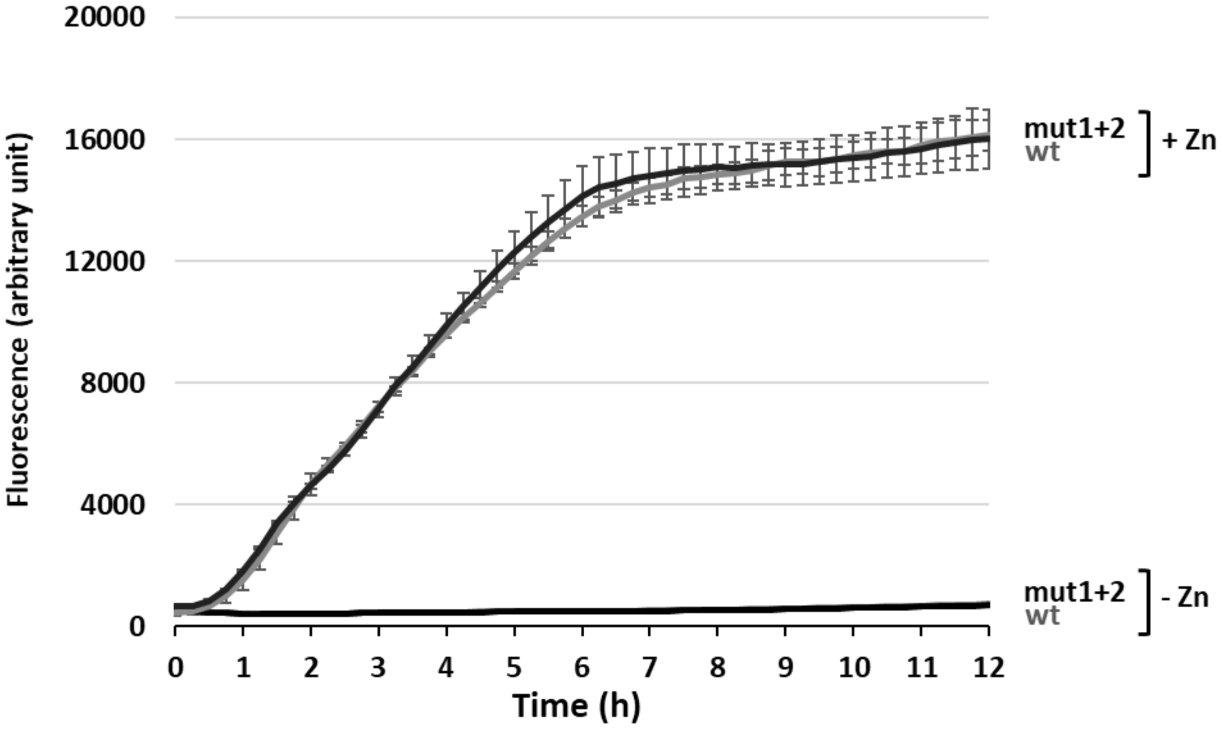

Figure S3: GFP promoter fusions containing 542 bp upstream of the *czcC* DNA region including either the wt or the mutated Zur boxes (mut1+2). Fusions were transformed into the wt PAO1 strain. Cultures were incubated at 37°C with shaking in a microplate reader and fluorescence measurement was performed every 15 min after the addition of 2mM $ZnCl_2$, or without the addition of Zn, as indicated. The fluorescence values are normalized with cell density. Standard deviations of the triplicates are indicated.
