# Supplementary material for: A Zur-mediated transcriptional regulation of the zinc export system in Pseudomonas aeruginosa
Source: BMC Microbiol. 2023 Jan 9;23:6. doi: 10.1186/s12866-022-02750-4 (PMC9827704; doi:10.1186/s12866-022-02750-4)
Supplement: Supplementary file 4 — Additional file 4: Table S1. Strains and plasmids used in this study [file 12866_2022_2750_MOESM4_ESM.pdf]

Table S1: Strains and plasmids used in this study

	Strain or plasmid	Relevant characteristic(s)[a]	reference or source
P. aeruginosa	Wild type	PAO1 wild type	laboratory collection
	Δzur	PAO1 Δ*zur*	this study
	ΔczcRΔczcS	PAO1 Δ*czcR*Δ*czcS*	(1)
	ΔcadR	PAO1 Δ*copR*Δ*copS*	(2)
E. coli	DH5α	recA1, endA1, hsdR17, deoR, thi-1, supE44, gyrA96, relA1, Δ(lacZYA-argF), U169(ϕ80dlacZΔM15)	(3)
	BL21(DE3)	E. coli str. B F– ompT gal dcm lon hsdSB(rB–mB–) λ(DE3 [lacI lacUV5-T7p07 ind1 sam7 nin5]) [malB+]K-12(λS)	(4)
**Plasmids**	pME3087	Suicide plasmid, Co1E1 replicon; Tc[r]	(5)
	pMMB66EH	Expressing vector carrying an IPTG-inducible promoter; Ap[r], Cb[r]	(6)
	pMMB66EH-*zur6HIS*	pMMB66EH derivative , carrying the *zur*gene fused with the C-terminal 6HIS-tag ; Ap[r], Cb[r]	this study
	pBBR1-*gfp*	Transcriptional *gfp* fusion cloning vector; Ap[r], Cb[r]	(7)
	pBBR1-*gfp* #1	pBBR1 derivative , carrying the short *czcR* promoter; Ap[r], Cb[r]	this study
	pBBR1-*gfp* #2	pBBR1 derivative , carrying the long *czcR* promoter; Ap[r], Cb[r]	this study
	pME6001-*czcRS*	pME6000 derivative , carrying the *czcR-czcS* operon under the *czcR* promoter; Gm[r]	this study
	pGex-2T-*zur*	GST-fusion expression plasmid,carrying the *zur* gene; Ap[r]	this study

[a]Antibiotic resistance are indicated by r: Tc: tetracycline, Ap: ampicillin, Cb: carbenicillin, Gm: gentamicin.

1. Caille, O., Rossier, C. and Perron, K. (2007) A copper-activated two-component system interacts with zinc and imipenem resistance in *Pseudomonas aeruginosa*. *J Bacteriol*, **189**, 4561-4568.
2. Ducret, V., Gonzalez, M.R., Leoni, S., Valentini, M. and Perron, K. (2020) The CzcCBA Efflux System Requires the CadA P-Type ATPase for Timely Expression Upon Zinc Excess in *Pseudomonas aeruginosa*. *Front Microbiol*, **11**, 911.
3. Sambrook, J., and D. W. Russell. . (2001.) *Molecular cloning: a laboratory manual* 3rd ed.Cold Spring Harbor Laboratory Press, Cold Spring Harbor, NY.
4. Studier, F.W., Rosenberg, A.H., Dunn, J.J. and Dubendorff, J.W. (1990) Use of T7 RNA polymerase to direct expression of cloned genes. *Methods in enzymology*, **185**, 60-89.
5. Voisard, C., Bull, C.T., Keel, C., Laville, J., Maurhofer, M., Schnider, U., Défago, G. and Haas, D. (1994), *Molecular Ecology of Rhizosphere Microorganisms*, pp. 67-89.
6. Furste, J.P., Pansegrau, W., Frank, R., Blocker, H., Scholz, P., Bagdasarian, M. and Lanka, E. (1986) Molecular cloning of the plasmid RP4 primase region in a multi-host-range tacP expression vector. *Gene*, **48**, 119-131.
7. Ouahrani-Bettache, S., Porte, F., Teyssier, J., Liautard, J.P. and Kohler, S. (1999) pBBR1-GFP: a broad-host-range vector for prokaryotic promoter studies. *Biotechniques*, **26**, 620-622.
