# Supplementary material for: A Zur-mediated transcriptional regulation of the zinc export system in Pseudomonas aeruginosa
Source: BMC Microbiol. 2023 Jan 9;23:6. doi: 10.1186/s12866-022-02750-4 (PMC9827704; doi:10.1186/s12866-022-02750-4)
Supplement: Supplementary file 5 — Additional file 5: Table S2. Primers used in this study [file 12866_2022_2750_MOESM5_ESM.pdf]

Table S2: Primers used in this study

			EMSA		
Amplicon	PA number	Primer	Sequence 5'-3'	Position (from ATG)	Lenght
pczcR	PA2523	686	GCCGGTACCACTTCGGCAACCTTCGAAGAG	-302	301
		687	GCCAGATCTGTTCGCCCCTATATAAAGTA	-1	
znuB	PA5501	676	CACCTCGCTGCTGATCATTC	576	190
		677	AAAACTCAGCAGGAACAGGC	765	
			RT-qPCR		
Amplicon	PA number	Primer	Sequence 5'-3'	Position (from ATG)	Lenght
czcR	PA2523	419	GTCATCACCCGGACGCAGATCAT	502	153
		420	GTAGCCGACGCCGCGAATGGTAT	654	
czcC	PA2522	czcC1	GGTCAGCATCGGCAGCAAGTACG	834	206
		czcC2	GGTCGTAGGCCTGTACCGCTTCG	1039	
czcD	PA0397	574	GGCGTGGCCTTCTATATCCT	285	183
		575	TCCAGACCTCCAGGTAGGC	450	
cadA	PA3690	1086	CATCAACGCCCTGATGAGTA	543	231
		1087	GCTTCCAGTTCCACTTGCTT	773	
oprF	PA1777	594	GGTTACTTCCTGACCGACGA	172	209
		595	TCGCTGTTGATGTTGGTGAT	380	
			DNA cloning		
	Amplicon	Primer	Sequence 5'-3'	Lenght	
Footprinting	*pczcR* 5'FAM	1271	FAM-ACTTCGGCAACCTTCGAAGAG	353	
		857	GGTGCAGGTAGTCGGCAGTC		
	pczcR 3'FAM	686	ACTTCGGCAACCTTCGAAGAG	353	
		1272	FAM-GGTGCAGGTAGTCGGCAGTC		
GFP fusions	*pczcR*#1	686	GCCggtaccACTTCGGCAACCTTCGAAGAG	301	
		687	GCCagatctGTTCGCCCCTATATAAAGTA		
	pczcR#2	895	CGCggtaccGGAACCACGCAACCGTTCAT	457	
		687	GCCagatctGTTCGCCCCTATATAAAGTA		
Mutant	*Δzur*	678	CGgaattcCATGGTCGTGCTCGTCGTG	510	
		679	caccagcgcgttgtccatcaggcgtccttctggtcGTGGTCATGGGGCTGGCA		
		680	GACCAGAAGGACGCCTGA	500	
		681	CGggatccGTAGAGTTCAGCCTGGCC		
pMMB66EH-zur6HIS	*zur*	731	GCCgaattcATGTACAAGATTGCGCCC	500	
		732	GCCggatccTCAatgatgatgatgatgatgGGCGTCCTTCTGGTCCC		
pME6001-czcRS	*czcRS*	384	GGGctcgagTCTGCTGATCGTCGTCGGCG	2963	
		385	GGGaagcttGTTCTTCTCGCTGCCTGTTC		
pGEX2T-zur	*zur*	1044	GCGggatccATGTACAAGATTGCGCCCAAGACCC	503	
		1045	GCCgaattcTCAGGCGTCCTTCTGGTCCC		
			5'RACE		
		Primer	Sequence 5'-3'		
		sp1R	GCAGGCCGTCGATGCCATCG		
		sp1C	TGCCGCGCGCCGCTGGCGAT		
		sp2R	GGTGCAGGTAGTCGGCAGTC		
		sp2C	GCCGCCAGCTCGGGGTTGCT		
		sp3R	AGTCTTGACTTCATCTTCGA		
